# Suppression of the fat storage defect of *C. elegans dbl-1/BMP* signaling mutants correlates with improved survival on a bacterial pathogen

**DOI:** 10.17912/micropub.biology.002067

**Published:** 2026-03-26

**Authors:** Katerina K Yamamoto, Alan Cantos, Daeun (Grace) Chun, Tiffany Cruz, Daniel Martinez, Cathy Savage-Dunn

**Affiliations:** 1 Biology, Queens College, CUNY, New York, NY, US; 2 PhD Program in Biology, CUNY Graduate Center, New York, NY, US

## Abstract

Innate immunity is a fundamental form of defense that is regulated by conserved signaling pathways. One such pathway is the DBL-1 /BMP (Bone Morphogenetic Protein) pathway, which is known to have a role in the immune response in the nematode *Caenorhabditis elegans .* DBL-1 /BMP signaling also regulates lipid metabolism and total fat stores. We recently demonstrated that BMP-dependent regulation of fatty acid metabolism contributes to host survival on the bacterial pathogen *Serratia marcescens .* We further hypothesized that suppressors of the low-fat phenotype of *dbl-1* would have better survival against the bacterial pathogen *S. marcescens* . We isolated such suppressors in a forward genetic screen following EMS mutagenesis of *dbl-1 ( wk70 )* mutants. The *dbl-1* suppressor strains CS722, CS742, and CS751 were tested on *S. marcescens* , using *dbl-1* mutant and wild-type strains as controls. We observed that the suppressor strains did indeed survive longer against *S. marcescens* than the *dbl-1* mutants. Longer survival was correlated with a higher-fat phenotype, once again reiterating the correlation between neutral lipid levels and host-pathogen response.

**Figure 1. Suppressors of dbl-1 low-fat phenotype also suppress poor survival on S. marcescens .** (A) Fourth larval stage (L4) animals stained by Oil Red O (ORO) show that the suppressor strains CS722, CS742 and CS751 are higher-fat than *dbl-1* mutants. The scale bar shows 100 μm. (B) Quantified lipid accumulation of wild-type (WT), *dbl-1* and the suppressor strains, stained by ORO at L4. ORO experiments were conducted with at least 20 animals per genotype. Brown-Forsythe and Welch ANOVA multiple comparisons tests were used to determine significance. Boxes show second and third quartiles; whiskers show minimum and maximum values. ns = *P* > 0.05; ** = *P* ≤ 0.01; **** = *P* ≤ 0.0001. (C) Survival analysis of wild-type animals, *dbl-1* mutants and CS722 suppressor on *S. marcescens* bacteria. *n* values: wild type (70), *dbl-1* (95), CS722 (85). One representative trial of three replicates shown. ns = *P* > 0.05; **** = *P* ≤ 0.0001. Black denotes significance relative to wild-type control; red denotes significance relative to *dbl-1 .* (D) Survival analysis of wild-type animals, *dbl-1* mutants and CS742 suppressor on *S. marcescens* bacteria. *n* values: wild type (86), *dbl-1* (79), CS742 (76). One representative trial of three replicates shown. ns = *P* > 0.05; **** = *P* ≤ 0.0001. Black denotes significance relative to wild-type control; red denotes significance relative to *dbl-1 .* (E) Survival analysis of wild-type animals, *dbl-1* mutants and CS751 suppressor on *S. marcescens* bacteria. *n* values: wild type (70), *dbl-1* (95), CS751 (67). One representative trial of three replicates shown. ns = *P* > 0.05; **** = *P* ≤ 0.0001. Black denotes significance relative to wild-type control; red denotes significance relative to *dbl-1 .
*

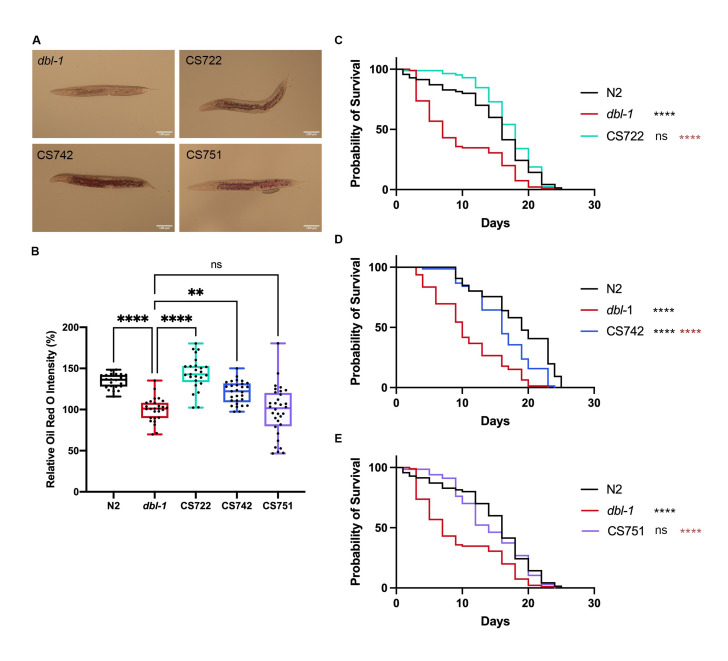



## Description

The DBL-1 /BMP signaling pathway was first identified for its roles in body size regulation, male tail patterning, and mesodermal lineage development. Later, it was shown to regulate innate immunity and fat metabolism in *C. elegans* (Gumienny & Savage-Dunn, 2013). The pathway begins with the secreted DBL-1 ligand binding a heterotetrameric complex of SMA-6 and DAF-4 , the type I and type II receptors, respectively. Signal is then transduced by the receptor-regulated Smads SMA-2 and SMA-3 , and the common mediator Smad SMA-4 . The complex then enters the nucleus to work with transcription factors to regulate gene expression. The pathway was initially linked to immune response when *dbl-1* mutants showed reduced survival after exposure to *S. marcescens* (Mallo et al., 2002). Exposure to other bacterial and fungal pathogens also results in decreased survival, demonstrating that DBL-1 /BMP signaling affects a broad range of immune challenges (Ciccarelli, Bendelstein, et al., 2024; Ciccarelli, Wing, et al., 2024; Portal-Celhay et al., 2012; So et al., 2011; Tenor & Aballay, 2008; Zugasti & Ewbank, 2009). These studies revealed that the DBL-1 pathway induces the expression of antimicrobial peptide genes upon pathogen exposure.

In addition to its role in innate immunity, DBL-1 /BMP signaling regulates fat storage, as BMP mutants consistently show reduced neutral lipid levels compared to wild type (Clark et al., 2018; Yu et al., 2017). We previously explored whether this DBL-1 /BMP regulation of lipid metabolism was contributing to the organism's pathogen response. We found that pathogen exposure alters fat storage, lipid droplet mobilization and fatty acid metabolism in a DBL-1-dependent manner (Yamamoto et al., 2025). These findings suggest a link between host fatty acid metabolism and survival upon pathogen exposure that beckons further study.

As part of our interest in the regulation of fat metabolism by DBL-1 , we conducted a genetic screen for suppressors of the low-fat phenotype of *dbl-1* mutants. Independently isolated suppressor strains CS722, CS742, and CS751 show clear differences in neutral lipid storage by Oil Red O (ORO) staining compared with *dbl-1* mutants ( Figure 1A, B). These mutants are unlikely to be simple revertants of the *wk70* nonsense mutation, because they still display the small body size phenotype, though this conclusion has not been verified by sequencing. The *dbl-1* mutants exhibited low fat accumulation, as expected. CS722 and CS742 each displayed a noticeable and statistically significant increase in staining intensity, showing a full or partial restoration of neutral lipid storage relative to the *dbl-1* baseline. CS751 did not have an increase in mean staining intensity, but did have a higher maximum intensity compared with *dbl-1* mutants as well as some visibly larger lipid droplets. Interestingly, none of the isolated suppressor mutants resulted in suppression of the small body size defect, indicating that these two phenotypes are separable. Our long-term goal is to determine the causative genetic changes and the mechanisms of suppression in these strains. Nevertheless, we reasoned that these strains could be employed to probe in an independent, unbiased manner the relationship between DBL-1 regulation of lipid metabolism and its support of host survival on a pathogen. Specifically, we asked whether selecting for changes in fat accumulation is associated with changes in the unselected phenotype of pathogen survival.

To determine whether the candidate suppressor strains also modify the pathogen sensitivity associated with a *dbl-1* mutant, we performed survival assays under pathogenic conditions using *Serratia marcescens .* CS722 showed a significant improvement in survival relative to *dbl-1* (p < 0.0001), while demonstrating no statistically significant difference compared to N2 ( Figure 1C ). Thus, CS722 is capable of restoring both lipid storage and pathogen resistance similar to wild-type levels. CS742 showed a statistically significant improvement in survival relative to *dbl-1* (p < 0.0001), although it did not reach the level of resistance of N2 ( Figure 1D ). CS742 thus showed a partial restoration of survival relative to *dbl-1* **.** Lastly, CS751 showed a significant improvement from *dbl-1* (p < 0.0001), while having no significant difference from N2 ( Figure 1E ). This result suggests that CS751 can act as a genetic suppressor of the *dbl-1* survival defect. In all three survival assays, *dbl-1* showed significantly reduced survival compared to N2 (p < 0.0001) as expected, due to its role in innate immune function in *C. elegans .* Overall, all three independent suppressor strains restored lipid storage relative to *dbl-1* and significantly improved survival under pathogenic conditions. We conclude that pathogen resistance, unlike body size, is correlated with the DBL-1-dependent regulation of lipid metabolism.

In summary, we tested whether regulation of lipid metabolism influences survival of *C. elegans* during infection with the bacterial pathogen *Serratia marcescens* . Across all trials, we observed that all three selected *dbl-1* suppressor strains consistently survived longer on the pathogen. This finding suggests that genetic alterations that modify lipid levels without necessarily restoring them to wild-type can still confer a measurable survival advantage. Our results support a model in which lipid metabolic state contributes directly to pathogen resistance.

*C. elegans* relies on innate immunity and behavioral avoidance to combat infection, as it lacks an adaptive immune system. Recent literature shows that *C. elegans* may also be relying on changes to lipid metabolism with pathogen exposure triggering lipolysis and depletion of lipid droplets in a pathogen-specific manner, regulated by NHR-49 (Dasgupta et al. 2020). Another study supported NHR-49 's involvement, finding that its pathogen-induced targets included lipid metabolism genes (Naim et al., 2021). Another transcription factor, SKN-1 , has also been shown to regulate lipids after pathogen exposure (Nhan et al., 2019). Specific lipid species, such as oleic acid and two 18-carbon polyunsaturated fatty acids, are also required for host survival during infection (Anderson et al., 2019; Nandakumar & Tan, 2008). Our recent work demonstrated that *C. elegans* undergo BMP-dependent changes to lipid metabolism after exposure to *S. marcescens* , specifically changes to β-oxidation and fatty acid desaturation gene expression (Yamamoto et al., 2025).

The DBL-1 /BMP signaling pathway, which controls body size, lipid metabolism, and innate immunity, was originally identified as an immune regulator because mutants show decreased survival on pathogens such as *S. marcescens* (Gumienny & Savage-Dunn, 2013; Mallo et al., 2002). Our findings build on this earlier work by showing that a suppressor of the *dbl-1* low-fat phenotype displays improved survival, suggesting that altered lipid regulation in this background contributes to enhanced pathogen tolerance or resistance.

Together, these results reinforce an emerging idea that lipid metabolism is a component of an effective immune response in *C. elegans* . Further work is required to determine which specific metabolic genes are mediating this effect in the suppressors, and whether the suppressor mutation interacts with known regulators such as NHR-49 or SKN-1 . Further work is also required to identify to what extent this regulatory interaction is conserved.

## Methods

***C. elegans* Strains and Growth Conditions.** *C. elegans* strains were grown on EZ worm plates containing streptomycin (550 mg Tris-Cl, 240 mg Tris- OH , 3.1 g Bactopeptone, 8 mg cholesterol, 2.0 g NaCl, 200 mg streptomycin sulfate, and 20 g agar per liter) to be consistent with previous studies from the lab (Ciccarelli, Bendelstein, et al., 2024; Clark et al., 2018). All strains were maintained on E. coli DA837 , a commonly used streptomycin resistant variant of OP50 , at 20°C. N2 was used as a wildtype control in all experiments. Suppressor strains were isolated as described below. They are provisionally designated as CS722 *dbl-1 ( wk70 ); sup(qc51)* , CS742 *dbl-1 ( wk70 ); sup(qc71)* , and CS751 *dbl-1 ( wk70 ); sup(qc80)* pending mapping and further characterization.

**Bacteria.** Control bacteria used in all experiments was *Escherichia coli* strain DA837 , cultured at 37°C. The pathogen used for pathogenic bacteria exposure was *Serratia marcescens* strain Db11 (ATCC #13880) cultured at 37°C. All experiments involving pathogens were conducted on EZ worm plates without streptomycin.

**Suppressor Screen.** *dbl-1 ( wk70 )* is a nonsense allele in the *dbl-1* coding region. *dbl-1 ( wk70 )* mutants underwent EMS mutagenesis and F2 progeny were screened by BODIPY to identify suppressors of lipid metabolism, exhibiting a higher-fat phenotype than the low-fat *dbl-1* mutants. Candidate suppressors were re-screened by BODIPY, then validated by one trial of ORO. Full details of the screen will be described elsewhere.

**EMS Mutagenesis** . Animals were ready for mutagenesis when they reached L4 to young adult and were washed into a 15mL tube with M9. In a 50mL tube, 1mL of M9 and 20 μL of EMS were carefully mixed to make EMS working solution. The contents of the 15mL tube were poured into the EMS working solution container. The animals were left in the EMS working solution for 4 hours, mixing gently once per hour to resuspend the animals. After 4 hours, the mutagenized animals were washed once with M9, then transferred to the edge of a worm plate with bacteria. After crawling for 30 minutes on the plate, the animals were transferred to individual plates. These are the P0 animals. From the progeny of the P0's, F1 animals were transferred to plates containing BODIPY (see below), with two F1's per plate. The F2 progeny were then screened for fat content in the following generation. For a recessive mutation, we expected 1/8 animals on the plate to have a higher-fat phenotype. Proper safety precautions were taken for handling EMS including personal protective equipment, and inactivation of EMS with 1M Na OH .

**BODIPY.** BODIPY 558/568 C12 (Red) was diluted to a 5mM stock in DMSO. For use as a live stain, 5μM BODIPY working solution was prepared in PBS. On EZ worm plates, after the DA837 bacterial lawn has grown, 100μL BODIPY working solution was added per plate. The plates were gently shaken to disperse liquid across the surface. Once the plates dried, they were used. Live animals were placed on the plates, and their progeny were screened using a fluorescent dissecting microscope.

**Oil Red O (ORO) Neutral Lipid Staining.** Oil Red O (ORO) staining was done as previously described (Clark et al., 2018). ORO stock solution was prepared by dissolving 0.25 g ORO powder in 50 mL isopropanol. Animals were collected after the desired time of pathogen exposure in PCR tube caps and washed three times in PBS to remove excess bacteria. Worms were fixed for 1 hour in 60% isopropanol while rocking at room temperature with caps covered with PCR tubes. While worms were fixing, the ORO working solution was made and allowed to rock at room temperature for 1 hour. After the working solution had equilibrated for 1 hour, it was filtered using a 10 mL syringe through a 0.45 µm filter, then through a 0.2 filter. The 60% isopropanol was removed and replaced with ORO working solution. The caps were covered with tubes and left overnight to stain while rocking at room temperature. The next day, the ORO was removed, and worms were washed once with PBS with 0.01% Triton, and then left in PBS while preparing slides for imaging. Worms were mounted on 2% agar pads on glass slides and imaged on a Zeiss Axioscope 2 using a Gryphax camera with Gryphax software. Images were taken using a 10x objective. For every animal, a 50 pixel by 50 pixel region was selected for quantification, ensuring the region was anatomically consistent across all images.

**Survival Analysis.** Survival analysis was done as previously described (Yamamoto et al. 2025; Ciccarelli, Bendelstein, et al., 2024; Ciccarelli, Wing, et al., 2024). Each survival plate was seeded with 500 µL of Db11 . 50 µM 5-Fluoro2'-deoxyuridine (FUdR) was added to each plate to prevent progeny and reduce the incidence of matricide by internal hatching of embryos. All survival experiments were carried out at 20°C. For each genotype, 120 L4 animals were picked for the experiment, and 20 animals plated per survival plate. The numbers of alive and dead animals were counted at least 4 days per week. During the experiment, some animals were lost due to burrowing, desiccation, etc. These animals were censored as their deaths were not observed. All survivals were repeated. Statistical analysis was done using the Log-rank (Mantel-Cox) test.

**Statistical Analysis.** Statistical analysis was performed in Graphpad Prism 10.

## Reagents

*Caenorhabditis elegans* Strains:

**Table:** 

Strain	Genotype	Source
N2	Wildtype (Bristol)	CGC
LT121	*dbl-1 ( wk70 ) *	CGC
CS722	*dbl-1 ( wk70 ); sup(qc51) *	Savage-Dunn Lab
CS742	*dbl-1 ( wk70 ); sup(qc71) *	Savage-Dunn Lab
CS751	*dbl-1 ( wk70 ); sup(qc80) *	Savage-Dunn Lab

Bacterial Strains:

**Table:** 

Strain	Species	Source
DA837	*Escherichia coli*	CGC
Db11	*Serratia marcescens*	ATCC (#13880)
